# Clinically-defined preoperative serum phosphorus abnormalities and outcomes of coronary artery bypass grafting: Retrospective analysis using inverse probability weighting adjustment

**DOI:** 10.1371/journal.pone.0225720

**Published:** 2019-12-18

**Authors:** Jungchan Park, Kwan Young Hong, Jeong Jin Min, Eunjin Kwon, Young Tak Lee, Wook Sung Kim, Hye Seung Kim, Kyunga Kim, Jong-Hwan Lee

**Affiliations:** 1 Department of Anesthesiology and Pain Medicine, Samsung Medical Center, Sung Kyunkwan University School of Medicine, Seoul, Korea; 2 Department of Thoracic and Cardiovascular Surgery, Samsung Medical Center, Sungkyunkwan University School of Medicine, Seoul, Korea; 3 Statistics and Data Center, Research Institute for Future Medicine, Samsung Medical Center, Seoul, Korea; 4 Department of Digital Health, SAIHST, Sungkyunkwan University, Seoul, Korea; Azienda Ospedaliero Universitaria Careggi, ITALY

## Abstract

**Background:**

Serum phosphorus is a well-known marker of vascular calcification, but the effects of serum phosphorus abnormalities defined by clinical criteria on the outcomes of coronary artery bypass grafting (CABG) remain unclear. We aimed to evaluate whether preoperative serum phosphorus abnormalities defined based on clinical criteria are associated with outcomes of CABG using a relatively new statistical technique, inverse probability weighting (IPW) adjustment.

**Methods:**

From January 2001 to December 2014, 4,989 consecutive patients who underwent CABG were stratified into normal (2.5–4.5 mg/dl; n = 4,544), hypophosphatemia (<2.5 mg/dl; n = 238), or hyperphophatemia (>4.5 mg/dl; n = 207) groups depending on preoperative serum phosphorus level.

**Results:**

The primary outcome was all-cause death during a median follow-up of 48 months. Secondary outcomes were cardiovascular death, graft failure, myocardial infarction, repeat revascularization, and stroke. In multivariate Cox analysis, preoperative hypophosphatemia was significantly associated with all-cause death (hazard ratio [HR] 1.76; 95% confidence interval [CI] 1.13–2.76; P = 0.01). However, this association varied depending on chronic kidney disease and emergent operation (p for interaction = 0.05 and 0.03, respectively). In addition, analysis after IPW adjustment demonstrated that preoperative serum phosphorus abnormalities were not significantly associated with all-cause death (P = 0.08) or any secondary outcomes except graft failure. Graft failure was significantly associated with preoperative hypophosphatemia (HR 2.51; 95% CI 1.37–4.61; P = 0.003).

**Conclusion:**

Our study showed that preoperative serum phosphorus abnormalities in clinical criteria were not associated with outcomes after CABG except for graft failure. And, the association of hypophosphatemia with graft failure remains to be evaluated.

## Introduction

Serum phosphorus plays a pivotal role in various biologic processes including energy production, membrane transport, and cellular signaling [1]. Serum phosphorus levels are tightly regulated via dietary absorption, bone formation, and renal excretion, and thus directly reflect renal dysfunction and vascular calcification [2,3]; therefore, patients with renal dysfunction and cardiovascular disease typically show a graded association between serum phosphorus and poor prognosis [4–7].

In addition to the well-known adverse effects of serum phosphorus abnormalities, an increase in morbidity after cardiovascular surgeries has also been reported [8–10]. However, in contrast to the results of percutaneous coronary intervention [11,12], previous studies have shown an inconsistent relationship between serum phosphorus abnormalities and coronary artery bypass grafting (CABG) outcomes [8,13]. In addition, to the best of our knowledge, no prior studies have used the clinical criteria definition of phosphorus abnormalities to perform a direct three-group comparison of the outcomes of CABG among normal, hypo- and hyperphosphatemia groups.

Considering that serum phosphorus is a well-known marker of vascular calcification and an assay is readily available, the effect of preoperative serum phosphorus abnormalities defined based on clinical criteria on the outcomes of CABG need to be re-evaluated. Therefore, in the present study, we evaluated whether preoperative serum phosphorus abnormalities are related to outcomes of CABG using the definition of serum phosphorus abnormalities from clinical criteria and a relatively new statistical technique, inverse probability weighting (IPW) adjustment.

## Materials and methods

### Study population and data collection

This study was approved by the Institutional Review Board of Samsung Medical Center (IRB No. 2017-10-109-003) and conducted in accordance with the principles of the Declaration of Helsinki. Considering the nature of this retrospective study and the minimal risk to patients, the need for informed consent was waived by the institutional review board. From January 2001 to December 2014, a consecutive 5,456 adult patients who underwent CABG at our institution were enrolled initially. After excluding 467 patients without preoperative phosphorus data, a total of 4,989 patients were analyzed. Serum phosphorus levels were measured during preoperative evaluation (Roche/Hitachi cobas c 701/702, Mannheim, Germany). The normal range for phosphorus was provided by the manufacturer (2.5 to 4.5 mg/dl). The patients were stratified into three groups according to serum phosphorus levels (i.e., normal, hypophosphatemia, and hyperphosphatemia).

Perioperative medical data were collected using a standardized form and protocol. Perioperative medications, laboratory findings, and echocardiographic data were extracted automatically from electronic medical records with the aid of the Institutional Medical Information Department. Death records were collected from the national database. Other postoperative clinical outcomes and causes of death were collected though manual review of each case by independent researchers who were blinded to perioperative medical data including preoperative phosphorus level.

### Study outcomes and definitions

Clinical outcomes were defined according to a report on cardiovascular events in clinical trials from the American College of Cardiology Foundation/American Heart Association task force [14]. The primary outcome was all-cause death within a median follow-up of 48 months. Secondary outcomes were cardiovascular death, graft failure, and a composite of major adverse cardiovascular and cerebral events (MACCE), including all-cause death, myocardial infarction (MI), repeat revascularization, and stroke. Cardiovascular death was defined as death related to MI, cardiac arrhythmia, heart failure, stroke, or any other vascular causes. Graft failure was confirmed by coronary angiogram and defined as stenosis more than 50%, complete occlusion of the graft and/or an anastomosed coronary artery, or by string sign. Recurrent MI was defined as recurrent symptoms with new electrocardiographic changes compatible with MI or cardiac marker elevation according to the Fourth Universal Definition of MI [15]. Repeat revascularization was defined as revascularization on either target or non-target vessel. Stroke was defined as a new ischemic or hemorrhagic lesion with a neurological deficit lasting longer than 24 hours.

### Statistical analysis

We used ANOVA or Kruskal-Wallis test to compare differences in baseline characteristics. Covariates with a univariable effect of P < 0.1 or clinically relevant coavarates were retained in multivariable analysis. The following were covariates for adjustment: male sex, age, diabetes, ejection fraction <40%, chronic kidney disease, old MI, acute coronary syndrome, left main artery disease, emergent operation, off-pump technique, valve-combined operation, and use of the right internal thoracic artery and the right gastroepiploic artery. To further reduce selection bias and maximize the study power while maintaining the balance in confounding factors between the three groups, we conducted rigorous adjustment for differences in baseline characteristics of patients using weighted Cox proportional hazards regression models with IPW [16]. According to this technique, weights for patients with hypo- and hyperphosphatemia were the inverse of the propensity score and weights for patients with normal phosphorus level were the inverse of 1 ± the propensity score. The reduction in the risk of outcome was compared using a stratified Cox regression model. Adjusted hazard ratios (HRs) with 95% confidence intervals (CIs) were reported. In addition, we performed subgroup analysis to reveal hidden interactions with male sex, chronic kidney disease, ejection fraction <40%, valve-combined operation, emergent operation, and the off-pump technique. Statistical analyses were performed with SAS version 9.4 (SAS Institute, Cary, NC) and R 3.5.1 (Vienna, Austria; http://www.R-project.org/). All tests were 2-tailed and P < 0.05 was considered statistically significant.

## Results

### Patient characteristics

The baseline characteristics of the three groups are summarized in Table 1. Of the enrolled 4,989 patients, 4,544 (91.1%) 238 (4.8%), and 207 (4.1%) were stratified into the normal, hypophosphatemia, and hyperphosphatemia groups, respectively (Table 1). Mean serum phosphorus levels were 3.5 (±0.5) mg/dl, 2.1 (±0.3) mg/dl and 5.1 (±0.6) mg/dl in each group. The three groups had significantly different baseline characteristics including male sex, age, diabetes, ejection fraction < 40%, chronic kidney disease, old MI, emergent operation, off-pump technique, valve-combined operation, and the use of the right gastroepiploic artery. After IPW adjustment, the balance between the three groups was improved and presented as the change in the average standardized absolute mean difference in S1 Fig.

**Table 1:** Baseline characteristics.

	Normal (N = 4544)	Hypophosphatemia (N = 238)	Hyperphosphatemia (N = 207)	P value
Serum phosphate, mg/dl	3.5 (±0.5)	2.1 (±0.3)	5.1 (±0.6)	<0.001
**Demographic variables**				
Male sex	3347 (73.7)	198 (83.2)	118 (57)	<0.001
Age, years	63.1 (±9.8)	65.1 (±9.6)	61.6 (±12.2)	0.002
**Previous history**				
Hypertension	2808 (61.8)	151 (63.5)	141 (68.6)	0.13
Diabetes	2022 (44.5)	109 (45.8)	116 (56.0)	0.005
Ejection fraction < 40%	699 (15.4)	53 (22.3)	56 (27.1)	<0.001
Dyslipidemia	1527 (33.6)	71 (29.8)	78 (37.7)	0.22
Stroke	681 (15.0)	40 (16.8)	29 (14.0)	0.68
Chronic kidney disease	211 (4.64)	32 (13.5)	52 (25.1)	<0.001
COPD	1416 (31.2)	63 (26.5)	55 (26.6)	0.46
Smoking	1416 (31.2)	63 (26.5)	55 (26.6)	0.13
PAOD	343 (7.6)	17 (7.1)	20 (9.7)	0.51
Old MI	550 (12.1)	37 (15.6)	35 (16.9)	0.04
ACS	2253 (49.6)	134 (56.3)	109 (52.7)	0.1
Carotid arterial disease	1034 (22.8)	48 (20.2)	52 (25.1)	0.46
**Previous treatment**				
Redo CABG	64 (1.4)	2 (0.8)	2 (1.0)	0.89
Thrombolysis	26 (0.6)	2 (0.8)	1 (0.5)	0.77
PCI	841 (18.5)	45 (18.9)	46 (22.2)	0.41
Antiplatelet	3865 (85.1)	198 (83.2)	177 (85.5)	0.72
Statin	1961 (43.2)	98 (41.2)	92 (44.4)	0.77
**Intraoperative parameters**				
LMD	797 (17.5)	40 (16.8)	24 (11.6)	0.08
3VD	3128 (48.8)	171 (71.9)	142 (68.6)	0.62
Emergent operation	123 (2.7)	21 (8.8)	12 (5.8)	<0.001
Off-pump technique	3797 (83.6)	180 (75.6)	142 (68.6)	<0.001
Valve combined	258 (5.7)	16 (6.7)	25 (12.1)	0.001
Anastomosis number	3.8 (±1.3)	3.8 (±1.4)	3.7 (±1.5)	0.72
LITA	4398 (96.8)	227 (95.4)	196 (94.7)	0.14
RITA	3821 (84.1)	196 (82.4)	163 (78.7)	0.1
RGEA	719 (15.8)	50 (21.0)	21 (10.1)	0.01
Radial	105 (2.3)	3 (2.6)	8 (3.9)	0.19
SVG	853 (18.8)	49 (20.6)	45 (21.7)	0.46

Values are presented as n (%) or mean (±SD). Abbreviations: COPD: chronic obstructive pulmonary disease; PAOD: peripheral arterial occlusive disease; MI: myocardial infarction; CABG: coronary artery bypass grafting; PCI: percutaneous coronary intervention; LMD: left main artery disease; LITA: left internal thoracic artery; RITA: right internal thoracic artery; RGEA: right gastroepiploic artery; SVG: saphenous vein graft

### Serum phosphorus abnormalities and clinical outcomes

The overall incidence of all-cause death was 4.1% (206/4989), with a 3.7% incidence in the normal group, 9.7% in the hypophosphatemia group, and 6.3% in the hyperphosphatemia group (P < 0.6). In multivariate Cox regression analysis only hypophosphatemia was significantly associated with the incidence of all-cause death (HR 1.76; 95% CI 1.13–2.76; P = 0.01) (Table 2). However, on further analysis using IPW adjustment, the association between hypophosphatemia and all-cause death was not significant (HR 1.52; 95% CI 0.89–2.61; P = 0.12). The incidence of graft failure was significantly higher in the hypophosphatemia group after adjustment using multivariate Cox regression analysis and IPW analysis (HR 2.14; 95% CI 1.22–3.75; P = 0.01, HR 2.51; 95% CI 1.37–4.61; P = 0.003, respectively).

**Table 2:** Clinical outcomes.

			Univariate analysis		Multivariate analysis		IPW analysis	
		n (%)	Unadjusted HR (95% CI)	P value	Adjusted HR (95% CI)	P value	Adjusted HR (95% CI)	P value
*All-cause death*		206 (4.1)		<0.001*				0.08*
	Normal	170 (3.7)	1		1		1	
	Hypophosphatemia	23 (9.7)	2.55 (1.65–3.94)	<0.001	1.76 (1.13–2.76)	0.01	1.52 (0.89–2.61)	0.12
	Hyperphosphatemia	13 (6.3)	1.28 (0.96–1.69)	0.09	1.10 (0.82–1.47)	0.54	0.55 (0.27–1.15)	0.11
*Cardiovascular death*		98 (2.0)		0.06*				0.54*
	Normal	83 (1.8)	1		1		1	
	Hypophosphatemia	7 (2.9)	1.60 (0.74–3.46)	0.23	1.03 (0.47–2.26)	0.95	1.24 (0.52–2.95)	0.63
	Hyperphosphatemia	8 (3.9)	1.44 (1.00–2.07)	0.05	1.18 (0.81–1.73)	0.39	0.62 (0.24–1.61)	0.32
*Myocardial infarction*		38 (0.8)		0.43*				0.38*
	Normal	34(0.7)	1		1		1	
	Hypophosphatemia	1 (0.4)	0.56 (0.08–4.05)	0.56	0.39 (0.05–2.89)	0.36	0.39 (0.05–2.82)	0.35
	Hyperphosphatemia	3 (1.4)	1.38 (0.76–2.49)	0.29	1.01 (0.54–1.88)		2.14 (0.47–9.87)	0.33
*Repeat revascularization*		192 (3.8)		0.43*				0.69*
	Normal	176 (3.9)	1		1		1	
	Hypophosphatemia	6 (2.5)	0.64 (0.28–1.44)	0.28	0.64 (0.28–1.45)	0.28	0.67 (0.25–1.83)	0.44
	Hyperphosphatemia	10 (4.8)	1.10 (0.80–1.52)	0.54	0.90 (0.64–1.26)	0.55	1.16 (0.52–2.61)	0.72
*Stroke*		201 (4.0)		0.21*				0.72*
	Normal	177 (3.9)	1		1		1	
	Hypophosphatemia	11 (4.6)	1.18 (0.64–2.17)	0.6	1.00 (0.54–1.86)	0.99	0.91 (0.46–1.82)	0.79
	Hyperphosphatemia	13 (6.3)	1.26 (0.95–1.67)	0.11	1.174 (0.87–1.58)	0.3	1.35 (0.63–2.90)	0.45
*MACCE*		573 (11.5)		0.001*				0.85*
	Normal	497 (10.9)	1		1		1	
	Hypophosphatemia	41 (17.2)	1.56 (1.13–2.14)	0.01	1.27 (0.92–1.75)	0.15	1.10 (0.74–1.63)	0.63
	Hyperphosphatemia	35 (16.9)	1.23 (1.04–1.46)	0.02	1.07 (0.89–1.280	0.46	1.09 (0.67–1.76)	0.73
*Graft failure*		157 (3.1)		0.04*				0.01*
	Normal	135 93.0)	1		1		1	
	Hypophosphatemia	14 (5.9)	1.97 (1.14–3.42)	0.02	2.14 (1.22–3.75)	0.01	2.51 (1.37–4.61)	0.003
	Hyperphosphatemia	8 (3.9)	1.13 (0.79–1.61)	0.5	1.11 (0.77–1.60)	0.59	1.22 (0.50–2.94)	0.66

Covariates include male sex, age, diabetes, ejection fraction < 40%, chronic kidney disease, old myocardial infarction, acute coronary syndrome, left main artery disease, emergent operation, off-pump technique, right internal thoracic arterial graft, and right gastroepiploic artery

*Comparison among three groups on ANOVA

**Fig 1:**
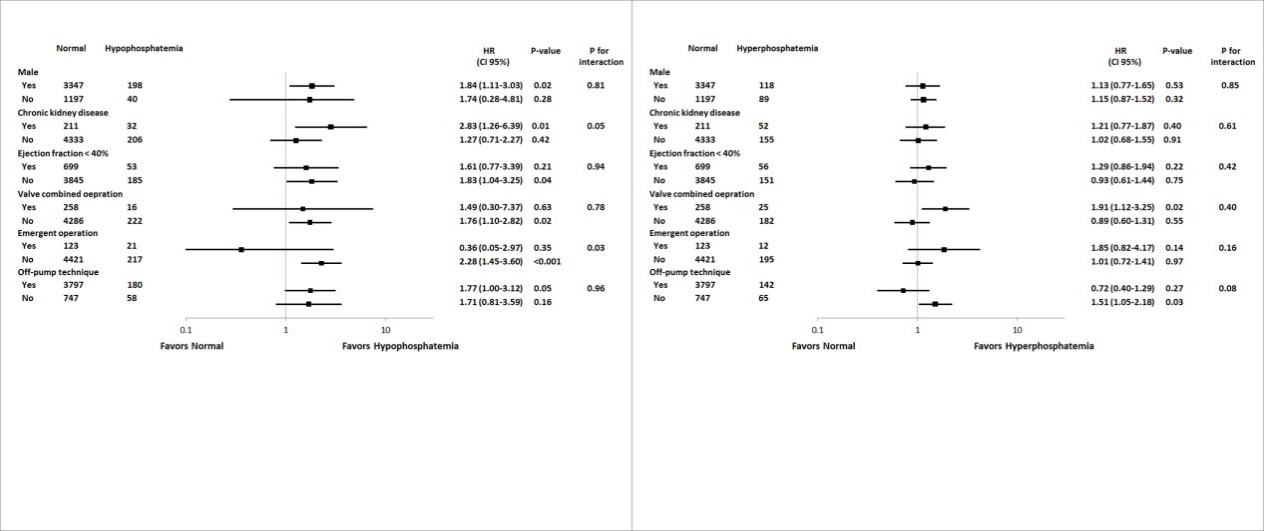
Subgroup analysis of all-cause death according to the following variables: male sex, chronic kidney disease, ejection fraction<40%, valve combined operation, emergent operation, and the off-pump technique.

**Fig 2:**
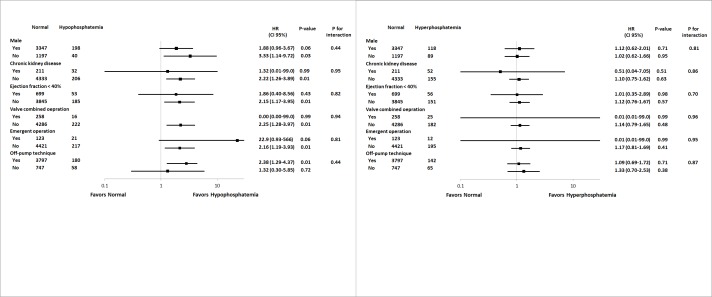
Subgroup analysis of graft failure according to the following variables: male sex, chronic kidney disease, ejection fraction<40%, valve combined operation, emergent operation, and the off-pump technique.

To determine whether the incidence of all-cause death and graft failure varied based on other covariates, we calculated the odds ratios (ORs) of various complex subgroups (Figs 1 and 2). Preoperative hypophosphatemia was associated with an increased incidence of all-cause death only in patients with chronic kidney disease or those undergoing non-emergent operation (P for interaction = 0.05 and 0.03, respectively). Other variables showed no interaction with all-cause death or graft failure.

## Discussion

In the present study, preoperative hypophosphatemia defined based on clinical criteria appeared to be associated with all-cause death and graft failure on univariate analysis. However, further analyses using IPW adjustment revealed that the association with all-cause death was not significant, and subgroup analysis indicated that this association varied based on chronic kidney disease and emergent operation. Interestingly, preoperative hypophosphatemia was independently associated with graft failure after CABG.

The relationship between serum phosphorus level and vascular calcification has been established based on a large body of evidence [2], and serum phosphorus abnormalities have been implicated in cardiovascular mortality and calcification of coronary arteries [4,5]. However, previous studies of patients undergoing coronary revascularization have shown inconsistent results [11–13]. Interestingly, all of these studies stratified patients into multiple groups in a graded manner by applying discrete criteria within normal range. Such methods might be convenient for showing statistical relationships but, in our opinion, does not reflect the clinical significance of phosphorus abnormalities. Moreover, the accuracy of the statistics applied in those studies to adjust covariates for comparing more than two groups is also questionable. Therefore, we stratified patients into normal, hypo-, and hyperphosphatemia groups using phosphorus levels defined based on clinical criteria and analyzed data using IPW adjustment.

Our study demonstrated that hyperphosphatemia is not associated with all-cause mortality. This result corresponds with the findings of a recent study on CABG [13]. The suggested explanation was that diseased arteries are fully replaced by endothelialized arteries or vein grafts in CABG, in contrast to other cardiovascular disease or coronary interventions, and so vascular calcification biomarkers are relatively useless in predicting outcomes [13]. On the other hand, in this study, hypophosphatemia appeared to be associated with all-cause death in multivariate analysis. Although this relationship has been previously suggested in open heart surgey [8], our subgroup analysis revealed that the association varied significantly based on other covariates and was limited to those with chronic kidney disease or those undergoing non-emergent operation. Considering that phosphorus-relevant biomarkers such as bone minerals, vitamin D, and parathyroid hormone are closely related to increases in cardiovascular risk, especially in the patients with kidney disease [7,17,18], these results might be related to the three main regulation mechanisms (i.e., diet intake, bone formation, and renal excretion), which interact dynamically. Poor oral intake is frequently observed in patients undergoing emergent procedures [19].

For adjustments among the three groups, we used a relatively new statistical method in this study. The propensity scores of relevant covariates are generally used to allow an observational study to mimic some of the particular characteristics of randomized trials [20] and there are four broad ways to use calculated propensity scores: matching, IPW, stratification, and covariate adjustment [21]. Although matching is the most frequently used method, using a logistic regression model to estimate the propensity score may lead to biased results in misspecified models [22]. Moreover, adequate adjustment with these methods could be challenging in comparisons among more than two groups. Therefore, using a generalized boosted regression has been proposed as an alternative approach for estimating propensity scores in IPW, and has also been suggested to be a suitable substitute matching method in some models [20]. For this study, institutional statistical experts participated in the design, and chose IPW as the most suitable statistical method to directly compare both hypo- and hyperphosphatemia to the normal group.

Interestingly, even after adjustment, hypophosphatemia was independently associated with graft failure. We suspect that this was related to endothelial dysfunction in hypophosphatemia [23]. In endothelial cells, nitric oxide is produced from L-arginine and molecular oxygen, and acts as a potent vasodilator that determines vascular remodeling [24]. However, in an experimental study, simulated hypophosphatemia decrease endothelial nitric oxide synthase activity and nitric oxide production via reduced intracellular calcium and increased protein kinase C β2 [23]. In addition, previous studies suggested that hypophosphatemia can cause a wide variety of cardiovascular diseases such as heart failure after cardiac surgery [8,25]. However, considering that graft failure after CABG can be caused not only by stenosis or thrombosis but also by native vessel flow or subclavian steal [26], further investigation of the relationship between hypophosphatemia and graft failure in CABG is needed.

There were several limitations to this study. First, since our study had a retrospective observational design, the possibility that hidden bias or unmeasured confounding factors might have influenced the results even after adjustment cannot be completely ruled out. Second, serum phosphorus abnormalities were determined based on a single preoperative measurement, and the duration of hypo- or hyperphosphatemia could not be considered in our analysis. Rapid turnover and tight regulation might have led to variation according to the timing of measurement. So, whether the correction of serum phosphorus level would improve clinical outcomes remains uncertain in this study and needs further investigation. Third, our study could not explain the underlying pathophysiologic mechanisms or determine whether phosphorus supplementation could improve outcomes. Finally, since we analyzed 14 years of data, our institutional protocols for surgical and perioperative management would have changed during the study period.

## Conclusions

In conclusion, preoperative serum phosphorus abnormalities defined based on clinical criteria were not associated with clinical outcomes of CABG. However, further studies will be needed to reveal the relationship between hypophosphatemia and graft failure.

## Supporting information

S1 Fig The change in the average standardized absolute mean difference before and after inverse probability weighting adjustment. (TIF)

S1 File Dataset. (XLSX)
