# Supplementary figures and images for: Clinically-defined preoperative serum phosphorus abnormalities and outcomes of coronary artery bypass grafting: Retrospective analysis using inverse probability weighting adjustment
Source: PLoS One. 2019 Dec 18;14(12):e0225720. doi: 10.1371/journal.pone.0225720 (PMC6919634; doi:10.1371/journal.pone.0225720)

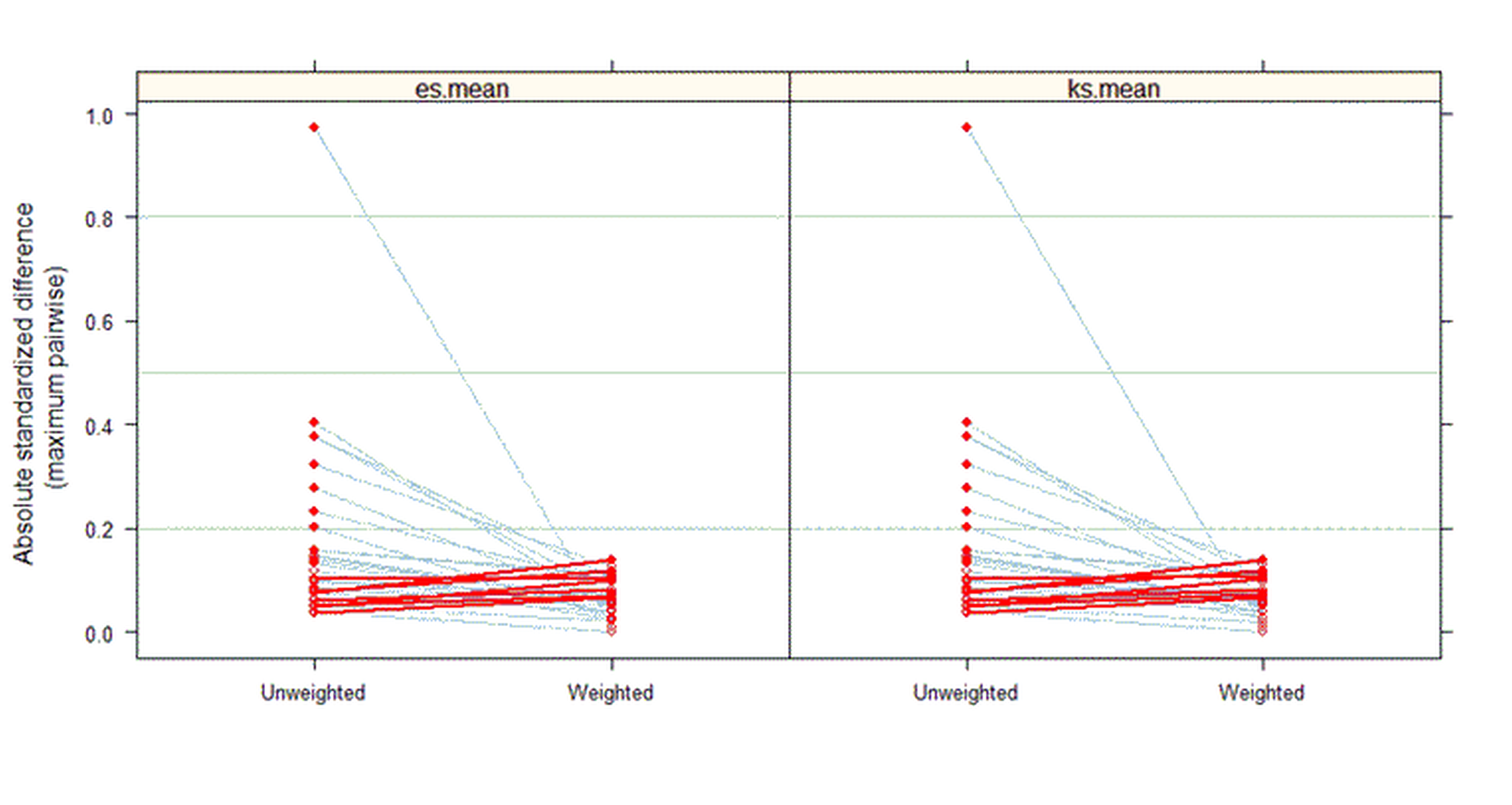

Supplement: S1 Fig — (TIF) [file pone.0225720.s001.tif]
